# Gamma-glutamyl transpeptidase (γ-GTP) has an ambivalent association with hypertension and atherosclerosis among elderly Japanese men: a cross-sectional study

**DOI:** 10.1186/s12199-019-0828-2

**Published:** 2019-11-30

**Authors:** Yuji Shimizu, Shin-Ya Kawashiri, Kairi Kiyoura, Kenichi Nobusue, Hirotomo Yamanashi, Yasuhiro Nagata, Takahiro Maeda

**Affiliations:** 1 Department of Community Medicine, Nagasaki University Graduate School of Biomedical Sciences, Nagasaki-shi, Sakamoto 1-12-4, Nagasaki, 852-8523 Japan; 2 Department of Cardiovascular Disease Prevention, Osaka Center for Cancer and Cardiovascular Disease Prevention, Osaka, Japan; 3 Department of Island and Community Medicine, Nagasaki University Graduate School of Biomedical Sciences, Nagasaki, Japan; 4 Department of General Medicine, Nagasaki University Graduate School of Biomedical Sciences, Nagasaki, Japan; 5 Center for Comprehensive Community Care Education, Nagasaki University Graduate School of Biomedical Sciences, Nagasaki, Japan

**Keywords:** Atherosclerosis, CD34+, Hypertension, γ-GTP

## Abstract

**Background:**

Even though there is bidirectional association between hypertension and atherosclerosis, atherosclerosis itself is involved in the process of endothelial repair. To clarify the association of endothelial repair with hypertension, a cross-sectional study was conducted.

**Methods:**

We conducted a cross-sectional study of 562 elderly Japanese men aged 60–69. As gamma-glutamyl transpeptidase (γ-GTP) could act as a marker of oxidative stress that injures endothelial cell and higher levels of CD34-positive cell indicate a higher activity of endothelial repair, we therefore performed a CD34-positive level specific analysis of γ-GTP on atherosclerosis and hypertension.

**Results:**

In the present study population, hypertension was independently and positively associated with atherosclerosis (multivariable odds ratio (OR) = 2.09 (1.30, 3.35)). Among participants with high CD34-positive cells, γ-GTP showed significant and positive association with atherosclerosis (OR of the log-transformed value of γ-GTP (OR) = 2.26 (1.32, 3.86)) but not with hypertension (OR = 0.77 (0.51, 1.17)). Among participants with low CD34-positive cells, even γ-GTP showed no significant association with atherosclerosis (OR = 0.92 (0.51, 1.68)), but was significantly and positively associated with hypertension (OR = 1.99 (1.27, 3.12)).

**Conclusions:**

γ-GTP revealed to have ambivalent association with hypertension and atherosclerosis. Active endothelial repair that is associated with atherosclerosis might have beneficial association with hypertension.

## Background

There is a bidirectional association between atherosclerosis and hypertension [1]; hypertension should be positively associated with atherosclerosis. However, atherosclerosis is one aspect of endothelial repair. Therefore, atherosclerosis per se might not be a major risk factor for hypertension, but age-related factor such as oxidative stress that causes progression of atherosclerosis might act as an independent risk factor both for hypertension and atherosclerosis.

The serum concentration of gamma-glutamyl transpeptidase (γ-GTP) could act as a marker of oxidative stress [2, 3], and oxidative stress has been reported to play a crucial role in the pathogenesis of both of atherosclerosis [4] and hypertension [5]. As aging is also known to be associated with oxidative stress [6, 7], γ-GTP could act as a marker of risk factor in age-related disease such as atherosclerosis and hypertension in elderly subjects.

Recent studies revealed a close connection between bone marrow activity and vascular maintenance activity (endothelial repair activity) [8–10]. As aging causes reduction in bone marrow function [11–13] and atherosclerosis is a well-known process because of aggressive endothelial repair activity, elderly subjects should have a lower capacity of maintenance of endothelium (endothelial repair) which results in a decrease of the progression of atherosclerosis. These studies indicate that the occurrence of atherosclerosis among elderly subjects partly indicates there is enough capacity for repair of the endothelium, which reduces the risk of hypertension. The CD34-positive cell is a source of progression for atherosclerosis as those cells could differentiate not only into an endothelial cell but also into a foam cell [14, 15]. Furthermore, our previous studies revealed that a higher level of CD34-positive cell (at and above median values) is necessary for the development of atherosclerosis [16, 17].

Therefore, we tested several hypotheses in this study. First, hypertension is positively associated with atherosclerosis. Second, a positive association between γ-GTP and atherosclerosis should be limited to subjects with a higher number of CD34-positive cells and consequently have a higher capacity of endothelium maintenance. Third, a positive association between γ-GTP and hypertension should be limited to subjects with low CD34-positive cells because those subjects might have a lower capacity for endothelial maintenance.

To examine these associations, we conducted a cross-sectional study of 562 elderly Japanese men aged 60–69 years who participated during their regular health check-up.

## Materials and methods

### Study population

The study population comprised 617 male residents aged 60–69 years, from Goto city and Saza town in the western part of Japan, who underwent an annual medical check-up from 2013 to 2015. The total number of male residents aged 60–69 in 2015 (estimated by the National Institute of Population and Social Security Research in March 2013) was 3264 for Goto city and 1010 for Saza town [18].

Participants without serum data (*n* = 38) and carotid intima-media thickness (CIMT) data (*n* = 1) were excluded from the present study population. Additionally, to avoid the influence of liver disease, participants with high aspartate transaminase (AST) (≥ 60 IU/L) and alanine transaminase (ALT) (≥ 60 IU/L) (*n* = 16) were also excluded. The remaining participants, comprising 562 men at a mean age of 65.5 years (standard deviation (SD) 2.6; age range 60–69), were enrolled in the study.

### Data collection and laboratory measurements

Trained interviewers obtained information on smoking and drinking status of the participants. After at least 5 min of rest in a sitting position, systolic and diastolic blood pressures on the right arm were recorded by using a blood pressure measuring device (HEM-907; Omron, Kyoto, Japan). Hypertension was defined as systolic blood pressure ≥ 140 mmHg and/or diastolic blood pressure ≥ 90 mmHg.

Body weight and height of participants, while wearing light clothing, were measured using an automatic body composition analyzer (BF-220; Tanita, Tokyo, Japan), and body mass index (BMI, kg/m^2^) was calculated as well.

Fasting blood samples were collected in a heparin sodium tube, a siliconized tube, and a sodium fluoride tube. Fresh blood samples (within 24 h from the drawing) from the heparin sodium tube were used to count the CD34-positive cells. The Beckton Dickinson Biosciences Trucount^TM^ technology, an accurate and reproducible single-platform assay cited in the International Society of Hematotherapy and Graft Engineering (ISHAGE) guidelines [19] and supported by automated software on the BD FACSCant^TM^ II system, was used to measure the count of circulating CD34-positive cells.

Serum triglycerides (TG), serum high-density lipoprotein cholesterol (HDLc), hemoglobin A1c (HbA_1C_), and γ-GTP contents were measured using standard laboratory procedures at SRL, Inc. (Tokyo, Japan).

CIMT was measured by ultrasonography of the left and right common carotid arteries, by an experienced vascular technician who used a LOGIQ Book XP with a 10-MHz transducer (GE Healthcare, Milwaukee, WI, USA). Maximum values for the left and right CIMT were calculated using automated digital edge-detection software (Intimascope; MediaCross, Tokyo, Japan) and a protocol that has been described in detail elsewhere [20]. The software allows automated identification of the edges of internal and external blood vessel membranes and automatically determines the distances at sub-pixel levels (estimated to be 0.01 mm in size, measuring CIMT with ten times higher axial resolution) using a polynomial measurement formula [21]. The right and left CIMT values that did not include plaque measurements were then calculated, and the maximum CIMT value (either the right or left) was used for further analysis. A previous study reported normal CIMT values as < 1.1 mm; we defined atherosclerosis as CIMT ≥ 1.1 mm [22].

### Statistical analysis

The characteristics of the study population for circulating CD34-positive cell levels (indicated by < 1.01 cells/μL, ≥ 1.01 cells/μL) were expressed as the mean ± SD for continuous variables and as the prevalence for habitual status (current drinker and current smoker) parameters. A trend test was performed using a regression model for mean values.

For the determination of the association between hypertension and atherosclerosis, logistic regression models were used to calculate the odds ratios (ORs) and 95% confidence intervals (CIs).

Circulating CD34-positive cell levels stratified by median value could act as a determinant factor on the association between atherosclerosis (CIMT) and variables [16, 17, 23, 24]. Moreover, those levels could act as a determinant factor in the association between hypertension and variables [25, 26]. Therefore, CD34-positive cell level-specific logistic regression models were used to calculate ORs and 95% CIs, to determine the associations between γ-GTP and atherosclerosis and between γ-GTP and hypertension.

Clarifying the endothelial activity status (CD34-posistive cell level)-specific association between oxidative stress, as evaluated by γ-GTP and hypertension, and the association between γ-GTP and atherosclerosis was the main objective of the present analysis. The factor that contributes to age-related bone marrow decline could act as a strong confounding factor in the present analysis because it could influence endothelial repair activity regardless of the endothelial condition. The factors that influence endothelial repair activity via the endothelial condition could not act as strong confounding factors but could act as associated factors in the present analysis. Therefore, two different models were used to adjust for confounding factors. In the first model, for the purpose of adjusting for the strong confounding factor, an adjustment was made only for age. The second model included other possible confounding factors known as classical cardiovascular risk factors to adjust for associated factors that were not strong confounding factors. For the analysis between hypertension and atherosclerosis, the confounding variables considered were BMI (kg/m^2^), smoking status (never smoker, former smoker, current smoker), alcohol consumption [never drinker, former drinker, current drinker (< 23 g/week, ≥ 23 g/week but < 46 g/week, ≥ 46 g/week but < 69 g/week, and ≥ 69 g/week)], HDLc (mg/dL), TG (mg/dL), and HbA1c (%). Four groups were made to classify the current drinkers. The first group had an alcohol consumption of lower than 23 g/week (< 23 g/week). The second group had an alcohol consumption of greater than or equal to 23 g/week but lower than 46 g/week (≥ 23 g/week but < 46 g/week). The third group had an alcohol consumption of greater than or equal to 46 g/week but less than 69 g/week (≥ 46 g/week but < 69 g/week). The last class had an alcohol consumption of greater than or equal to 69 g/week.

For the analysis between γ-GTP and atherosclerosis, the confounding variables considered were BMI (kg/m^2^), systolic blood pressure (mmHg), smoking status (never smoker, former smoker, current smoker), alcohol consumption [never drinker, former drinker, current drinker (< 23 g/week, ≥ 23 g/week but < 46 g/week, ≥ 46 g/week but < 69 g/week, and ≥ 69 g/week)], HDLc (mg/dL), TG (mg/dL), and HbA1c (%). For the analysis between γ-GTP and hypertension, the confounding variables considered were BMI (kg/m^2^), smoking status (never smoker, former smoker, current smoker), alcohol consumption [never drinker, former drinker, current drinker (< 23 g/week, ≥ 23 g/week but < 46 g/week, ≥ 46 g/week but < 69 g/week, and ≥ 69 g/week)], HDLc (mg/dL), TG (mg/dL), and HbA1c (%).

All statistical analyses were performed with the SAS system for Windows (version 9.4; SAS Inc., Cary, NC). Values of *p* < 0.05 were considered statistically significant.

## Results

### Clinical characteristics of the study population

Among the present study population, 239 subjects were diagnosed for hypertension and 89 for atherosclerosis. The median value of CD34-positive cell level is 1.01 cells/μL. Compared to participants with lower CD34-positive cell level (< 1.01 cells/μL), participants with higher CD34-positive cell level (≥ 1.01 cells/μL) showed significantly lower age while significantly higher BMI, TG, and HbA1c were observed (Table 1).

**Table 1:** Characteristics of the study population as per circulating CD34-positive cell levels

	CD34-positive cell levels		*p* values
	Low (< 1.01 cells/μL)	High (≥ 1.01 cells/μL)	
No. of participants at risk	280	282	
Age, years	65.7 ± 2.6	65.2 ± 2.6	0.030
Body mass index (BMI), kg/m^2^	22.7 ± 3.0	23.9 ± 2.8	< 0.001
Systolic blood pressure, mmHg	134 ± 18	134 ± 17	0.714
Diastolic blood pressure, mmHg	81 ± 12	81 ± 11	0.784
Current drinker, %	83.9	87.9	0.172
Current smoker, %	11.4	13.8	0.393
HDL cholesterol (HDLc), mg/dL	58 ± 14	56 ± 15	0.058
Triglycerides (TG), mg/dL	109 ± 82	125 ± 94	0.026
Hemoglobin A1c (HbA1c), %	5.6 ± 0.6	5.8 ± 0.7	0.001
Gamma-glutamyl transpeptidase (γ-GTP), IU/L	44 ± 38	50 ± 70	0.220
Carotid intima-media thickness (CIMT), mm	0.9 ± 0.2	0.9 ± 0.2	0.347

Values: mean ± standard deviation

### Association between atherosclerosis and hypertension

Hypertension was independently, significantly, and positively associated with atherosclerosis (Table 2).

**Table 2:** Odds ratios (ORs) and 95% confidence intervals (CIs) for atherosclerosis

	Hypertension		*p* values
	(−)	(+)	
No. at risk	323	239	
No. of cases (percentage)	38 (11.8)	51 (21.3)	
Age-adjusted ORs	1	2.02 (1.28, 3.20)	0.003
Multivariable ORs	1	2.09 (1.30, 3.35)	0.002

Multivariable ORs: adjusted further for age and BMI, alcohol consumption, smoking status, HDLc, TG, and HbA1C

*BMI* body mass index, *HDLc* HDL cholesterol, *TG* triglycerides, *HbA1c* hemoglobin A1c

### Association between atherosclerosis and γ-glutamyl transpeptidase (γ-GTP) as per circulating CD34-positive cell levels

Although no significant association between γ-GTP and atherosclerosis was observed in participants with lowcirculating CD34-positive cell counts, a significant positive association between those two variables was observed in participants with high circulating CD34-positive cell counts (Table 3).

**Table 3:** Odds ratios (ORs) and 95% confidence intervals (CIs) for atherosclerosis by circulating CD34-positive cell levels

	Gamma-glutamyl transpeptidase (γ-GTP)				*p* values	Log γ-GTP
	Q1 (low)	Q2	Q3	Q4 (high)		
Low CD34-positive cell						
No. at risk	78	74	52	76		
No. of cases (percentage)	15 (19.2)	13 (17.6)	11 (21.2)	8 (10.5)		
Age-adjusted ORs	1	0.90 (0.40, 2.04)	1.13 (0.47, 2.70)	0.50 (0.20, 1.26)	0.218	0.79 (0.48, 1.30)
Multivariable ORs	1	0.99 (0.41, 2.38)	1.18 (0.47, 3.01)	0.61 (0.21, 1.81)	0.520	0.92 (0.51, 1.68)
High CD34-positive cell						
No. at risk	60	68	89	65		
No. of cases (percentage)	6 (10.0)	8 (11.8)	12 (13.5)	16 (24.6)		
Age-adjusted ORs	1	1.12 (0.36, 3.44)	1.34 (0.47, 3.81)	2.91 (1.05, 8.05)	0.026	1.87 (1.17, 2.99)
Multivariable ORs	1	1.52 (0.46, 4.97)	1.77 (0.58, 5.41)	4.28 (1.34, 13.63)	0.013	2.26 (1.32, 3.86)

Multivariable ORs: adjusted further for age and BMI, alcohol consumption, smoking status, SBP, HDLc, TG, and HbA1C

*SBP* systolic blood pressure, *BMI* body mass index, *HDLc* HDL cholesterol, *TG* triglycerides, *HbA1c* hemoglobin A1c

### Associations between γ-glutamyl transpeptidase (γ-GTP) and two circulating CD34-positive cell level groups on atherosclerosis

Investigations of the associations between γ-GTP and the two CD34-positive cell groups (high level and low level) on atherosclerosis revealed a significant interaction. The age-adjusted *p* value for this interaction was *p* = 0.014 for age-adjusted model and *p* = 0.005 for the fully adjusted model.

### Association between hypertension and γ-glutamyl transpeptidase (γ-GTP) based on the circulating CD34-positive cell levels

Even though a significant positive association between γ-GTP and hypertension was observed in participants with low circulating CD34-positive cell counts, no significant positive association between those two variables were observed in participants with high circulating CD34-positive cell counts (Table 4).

**Table 4:** Odds ratios (ORs) and 95% confidence intervals (CIs) for hypertension by circulating CD34-positive cell levels

	Gamma-glutamyl transpeptidase (γ-GTP)				*p* values	Log γ-GTP
	Q1 (low)	Q2	Q3	Q4 (high)		
Low CD34-positive cell						
No. at risk	78	74	52	76		
No. of cases (percentage)	24 (30.8)	30 (40.5)	24 (46.2)	42 (55.3)		
Age-adjusted ORs	1	1.54 (0.79, 3.01)	1.93 (0.93, 3.99)	2.82 (1.45, 5.47)	0.002	1.89 (1.30, 2.75)
Multivariable ORs	1	1.57 (0.78, 3.17)	1.89 (0.88, 4.05)	2.84 (1.30, 6.22)	0.009	1.99 (1.27, 3.12)
High CD34-positive cell						
No. at risk	60	68	89	65		
No. of cases (percentage)	23 (38.3)	30 (44.1)	39 (43.8)	27 (41.5)		
Age-adjusted ORs	1	1.24 (0.61, 2.54)	1.24 (0.63, 2.42)	1.14 (0.56, 2.33)	0.743	0.91 (0.63, 1.31)
Multivariable ORs	1	1.46 (0.69, 3.09)	1.14 (0.55, 2.34)	1.02 (0.46, 2.28)	0.854	0.77 (0.51, 1.17)

Multivariable ORs: adjusted further for age and BMI, alcohol consumption, smoking status, HDLc, TG, and HbA1C

*BMI* body mass index, *HDLc* HDL cholesterol, *TG* triglycerides, *HbA1c* hemoglobin A1c

### Associations between γ-glutamyl transpeptidase (γ-GTP) and two circulating CD34-positive cell categories on hypertension

Investigations of the associations between γ-GTP and the two CD34-positive cell groups (high level and low level) on hypertension revealed a significant interaction. The age-adjusted *p* value for this interaction was *p* = 0.006 for age-adjusted model and *p* = 0.007 for the fully adjusted model.

## Discussion

The main findings of the present study are that among elderly participants with high CD34-positive cell, γ-GTP shows a significantly positive association with atherosclerosis but not with hypertension. Among elderly participants with low CD34-positive cell, no significant association between γ-GTP and atherosclerosis was observed but a significant positive association was observed between γ-GTP and hypertension.

Aging is a well-known process to promote the progression of atherosclerosis and hypertension [27, 28]. Our finding indeed revealed a significant positive association between atherosclerosis and hypertension.

Recent studies revealed a close connection between bone marrow activity and vascular maintenance activity (endothelial repair activity); bone marrow-derived endothelial progenitor cells, including CD34-positive cells, play an important role in vascular maintenance through endothelium repair [8–10]. As atherosclerosis is one aspect of endothelial repair and CD34-positive cell is known to be differentiated not only into an endothelial cell but also into a foam cell which is a known source of atherosclerosis [14, 15], therefore, circulating CD34-positive cell level could act as an indicator of endothelial activity [16, 17]. The high CD34-positive cell level indicates a higher activity of endothelial repair.

Furthermore, CD34-positive cell contributes to angiogenesis [29] that could compensate for the reduction of blood follow in the ischemic organ. Further, angiogenesis is necessary to develop atherosclerosis and also has an important function in the reduction of the ischemic condition by compensating for the blood flow  [30]. The ischemic condition is known to be associated with oxidative stress. As age-related physical change induces oxidative stress, elderly participants should have higher oxidative stress than younger participants. Elderly participants with developing atherosclerosis might have a higher capacity of reducing the oxidative stress than those without developing atherosclerosis as atherosclerosis promotes angiogenesis through the endothelial repair process.

Several studies indicated that bone marrow activity declines with advancing age [11–13]. Therefore, it is expected that the age-related decline of bone marrow activity might cause lower vascular maintenance activity (endothelial repair), which results in a lower presence of atherosclerosis.

As γ-GTP is reported to be associated with oxidative stress [2, 3] that is known to cause the age-related physical changes [6, 7], atherosclerosis [4], and hypertension [5], the γ-GTP level should correlate with the extent of endothelial injury and endothelial repair activity.

In the present study, our results indicate a significant positive association between γ-GTP and atherosclerosis in participants with high CD34-positive cell levels. As the presence of atherosclerosis among those participants indicates aggressive endothelial maintenance, atherosclerosis could act as an indicator of adjustment capacity for aging-related physical change. Therefore, no significant association between γ-GTP and hypertension was observed.

Although, the γ-GTP level should indicate the magnitude of endothelial injury that stimulates endothelial repair activity, sufficient values of endothelial repair could not be established in participants with the low CD34-positive cells. Thus, among participants with low CD34-positive cell, no significant association between γ-GTP and atherosclerosis was observed but a significant positive association between γ-GTP and hypertension was observed.

This study demonstrates the possible beneficial effects of atherosclerosis on hypertension which is caused by the age-related physical change. Therefore, this finding provides a novel diagnostic tool to indicate the mechanism of adaptation for age-related physical change among elderly participants.

Single-nucleotide polymorphism (SNP) in breast cancer suppressor BRCA1**-**related associated protein (BRAP) activates inflammatory cascades and increases the risk of carotid atherosclerosis [31] but reduces the risk of hypertension [32]. These studies also support the aforementioned possible beneficial effects of atherosclerosis on hypertension.

The clinical perspective of present study is that the absence of atherosclerotic lesion is not indicative of adequate endothelial maintenance.

This study has certain potential limitations that warrant further consideration. First, even though γ-GTP is reportedly associated with oxidative stress [2, 3], no data on oxidative stress was available for the participants included in our study. Further investigations in reactive oxygen species, superoxide dismutase, and catalases are necessary to elucidate the anti-oxidative stress mechanism related to atherosclerosis. Values of γ-GTP are known to be strongly influenced by alcohol consumption and the prevalence of current drinker is high in Japanese men and low in Japanese women [33]. Since there is no data on the levels of CD34-posistive cell in women, we could not evaluate the present associations for women. However, in the present study, the main results were essentially unchanged even after adjustment for confounding factors including alcohol consumption. As this was a cross-sectional study, causal relationships could not be established.

## Conclusion

In this study, we showed that among elderly participants with high CD34-positive cell, the γ-GTP level shows a significant positive association with atherosclerosis but not with hypertension. On the other hand, among elderly participants with low CD34-positive cell, no significant association between γ-GTP and atherosclerosis was observed, but a significant positive association between γ-GTP and hypertension was observed. Therefore, active endothelial repair that is associated with atherosclerosis could have beneficial effect on hypertension among elderly participants.

## Data Availability

The datasets generated during and/or analyzed during the current study are not publicly available due to ethical consideration but are available from the corresponding author on reasonable request.

## Abbreviations

ALT: Alanine transaminase
AST: Aspartate transaminase
BMI: Body mass index
CIMT: Intima-media thickness
HbA1c: Hemoglobin A1c
HDLc: High-density lipoprotein cholesterol
ISHAGE: International Society of Hematotherapy and Graft Engineering
OR: Odds ratio
SD: Standard deviation
TG: Triglycerides
γ-GTP: Gamma-glutamyl transpeptidase
